# Identification of Immunogenic Epitopes That Permit the Detection of Antigen-Specific T Cell Responses in Multiple Serotypes of Group B Coxsackievirus Infections

**DOI:** 10.3390/v12030347

**Published:** 2020-03-21

**Authors:** Ninaad Lasrado, Arunakumar Gangaplara, Rajkumar Arumugam, Chandirasegaran Massilamany, Sayli Pokal, Yuzhen Zhou, Shi-Hua Xiang, David Steffen, Jay Reddy

**Affiliations:** 1 School of Veterinary Medicine and Biomedical Sciences, University of Nebraska-Lincoln, Lincoln, NE 68503, USA; ninaad@huskers.unl.edu (N.L.); arunakumar.gangaplara@nih.gov (A.G.); raj.ar@huskers.unl.edu (R.A.); mchandirasegaran@gmail.com (C.M.); sxiang2@unl.edu (S.-H.X.); dsteffen1@unl.edu (D.S.); 2 Department of Statistics, University of Nebraska-Lincoln, Lincoln, NE 68503, USA; spokal@huskers.unl.edu (S.P.); yuzhenzhou@unl.edu (Y.Z.)

**Keywords:** coxsackievirus B, myocarditis, MHC-tetramers, T cells

## Abstract

Coxsackievirus group B (CVB) contains six serotypes that can affect various organs. Some of these organ-specific diseases such as myocarditis and pancreatitis can be caused by more than one serotype. Thus, development of immunological tools common to multiple serotypes is desired. This is especially critical for analyzing antigen-specific T cell responses at a single cell level. To this end, we made efforts to identify the immunogenic epitopes of CVB3 leading us to localize three T cell epitopes within the viral protein 1 (VP1) namely, VP1 681–700, VP1 721–740 and VP1 771–790. First, we confirmed their immunogenicity in the immunization settings. Second, we sought to verify the ability of VP1 epitopes to bind major histocompatibility complex (MHC) class II (IA^k^) molecules. Third, we created MHC class II (IA^k^) dextramers and tetramers and ascertained the T cell responses to be antigen-specific. Fourth, we analyzed the T cell responses in animals infected with CVB3 and noted the magnitude of antigen-specific T cell responses occurring in the order of VP1 721–740 and VP1 681–700 followed by VP1 771–790 as verified by proliferation assay and IA^k^ tetramer staining. All epitopes induced interferon (IFN)-γ as a major cytokine. Finally, we investigated whether the VP1 tools generated for CVB3 can also be used to verify T cell responses in infections caused by other serotypes. To this end, we established the CVB4 infection model in A/J mice and found that the CVB4 infection led to the induction of IFN-γ-producing T cell responses primarily for VP1 721–740 and VP1 681–700. Thus, the VP1-specific tools, particularly IA^k^ tetramers can be used to monitor anti-viral T cell responses in multiple CVB serotypes.

## 1. Introduction

Enteroviruses commonly cause infections in humans [1]. These are small (30 nm), non-enveloped, positive-sense, single-stranded RNA viruses possessing an icosahedral capsid consisting of 60 subunits bearing four structural viral proteins (VPs): VP1 to VP4. The viral RNA is 7.5 kilo bases long, and the coding region is encompassed by non-translated regions at both 5′ and 3′ ends. The genus *Enterovirus* includes group A and group B coxsackieviruses. Within these, several serotypes have been identified (23 in group A; six in group B). While some syndromes are caused only by group A coxsackieviruses, diseases like myocarditis and pancreatitis are caused mainly by group B coxsackieviruses (CVB) [1,2,3].

Within the group B coxsackieviruses, CVB3 is commonly implicated in the causation of myocarditis. CVB-reactive antibodies are found in ~50% of dilated cardiomyopathy (DCM) patients, while enterovirus genomic material can be detected in up to 70% patients [4,5,6,7,8], suggesting CVB can be an important trigger of myocarditis/DCM. Two phases of CVB3 infection have been described in the mouse models that resemble human disease [9]. These include a viral/acute myocarditis phase (up to 14–18 days postinfection) and a nonviral/chronic myocarditis phase (beyond 18 days) [9]. In contrast to CVB3, CVB4 is commonly linked with the development of type 1 diabetes (T1D) as evidenced by serology, virus-recovery, and polymerase chain reaction (PCR) analysis [10,11,12,13]. Experimentally, CVB3 and CVB4 infections in young (4- to 6-week) non-obese diabetic (NOD) mice have been shown to protect them from developing diabetes; in contrast, the same infections have been shown to augment the disease in mice older than 12 weeks [14,15,16]. An emerging theme suggests that enteroviral infections, in the face of preexisting insulitis, augment the development of diabetes through infiltration of bystander immune cells, leading to destruction of insulin-producing pancreatic β islets. However, in the absence of preexisting insulitis, virus infections favor the generation of regulatory T (Treg) cells that can suppress diabetes development [14,17].

Because CVB infections are strongly associated with myocarditis and pancreatitis, and both diseases can be triggered by more than one serotype, we sought to develop tools that allow us to determine antigen-specific T cell responses in infections caused by multiple CVB serotypes. These investigations led us to identify three epitopes within the CVB3 viral protein1 (VP1) that induce varied degrees of T cell responses. By generating major histocompatibility complex (MHC) class II/IA^k^ dextramers and tetramers, we demonstrate that both CVB3 and CVB4 infections can lead to the generation of interferon (IFN)-γ-producing, VP1-specific CD4 T cells, predominantly for VP1 721–740, suggesting that the VP1-specific tools, importantly, IA^k^ tetramers, can be used to analyze antigen-specific T cell responses in CVB infections.

## 2. Materials and Methods

### 2.1. Mice

Six to eight-week-old male and female A/J mice (H-2^a^) were procured from the Jackson Laboratory (Bar Harbor, ME, USA) and maintained according to the institutional guidelines of the University of Nebraska-Lincoln, Lincoln, NE. Approval for animal studies was granted by the Institutional Animal Care and Use Committee, University of Nebraska-Lincoln (protocol #: 1398, approved January 3, 2017). Euthanasia was performed using a carbon dioxide chamber as recommended by the Panel on Euthanasia, the American Veterinary Medical Association.

### 2.2. Peptide Synthesis and Immunization Procedures

An overlapping peptide library that included a total of 30 peptides of 20-mers was synthesized by 9-fluorenylmethyloxycarbonyl chemistry. In addition, bovine ribonuclease (RNase) 43–56 (VNTFVHESLADVQA), biotinylated hen egg lysozyme (HEL) 46–61 (YNTDGSTDYGILQINSR) (Neopeptide, Cambridge, MA) were also synthesized. All peptides were purified to be more than 90% by high-performance liquid chromatography, and their identities were confirmed by mass spectroscopy. Ultra-pure water was used to dissolve peptides and stored at −20 °C. For immunizations, VP1 peptides (VP1 681–700, VP1 721–740 and VP1 771–790) were individually emulsified in the complete Freund’s adjuvant (CFA) containing *Mycobacterium tuberculosis* H37RA extract to a final concentration of 5 mg/mL(Difco Laboratories, Detroit, MI, USA). The peptide (100 µg per animal) emulsions were administered subcutaneously into mice in the inguinal and sternum regions [18,19].

### 2.3. Virus Propagation and Infection

HeLa cells [American Type Culture Collection (ATCC), Manassas, VA, USA] were grown to 80% to 90% confluence in 75 cm^2^ flasks in EMEM supplemented with 10% fetal calf serum (FCS). After removing the medium, the adherent monolayer of cells were infected with CVB3 (Nancy strain, ATCC) or CVB4 (E2 strain, kind gift from Dr. Shubhada Bopegamage, Slovak Medical University, Slovak Republic) with multiplicity of infection 1 in EMEM containing no FCS. The virus was allowed to adsorb to the cells by incubating the flasks at 37 °C for 1 h with gentle intermittent rotations every 10 to 15 min. Maintenance medium containing 2% FCS was then added and incubation was continued. After confirming the cytopathic effect of virus, the infected culture flasks were subjected for three rounds of freezing and thawing and culture supernatants containing virus were harvested by centrifuging cell lysates at 6000 rpm for 10 min at 4 °C. The tissue culture infective dose (TCID_50_) values were then determined according to Reed–Muench method. The virus stocks were aliquoted and preserved at −80 °C until further use.

For infection studies, the virus stock was diluted in 1x phosphate-buffered saline (PBS) to contain 2000 to 10,000 TCID_50_/200 µl, and the inocula were administered into A/J mice intraperitoneally, whereas the control (uninfected) mice received only 1x PBS. Animals were housed in filter-top cages (two to three mice/cage) assembled with closed air-circulation. Cages containing the chow diet and waterers were changed once every 3 days until the termination of the experiment, and the animals had ad libitum access to food and water during the entire period of study. Animals were inspected twice a day, and body weights were taken daily. Additionally, alternative food- and fluid-source, trans gel diet (ClearH2O, Portland, ME, USA) were placed in the cage floor as needed. Hearts and pancreata were collected from the animals that died naturally. At termination (days, 16 to 21 post-infection), animals were euthanized using CO_2_ chamber prefilled with 2% CO_2_, and spleens, lymph nodes, hearts, and pancreata were collected.

### 2.4. Proliferative Response

Lymph node cells (LNCs) and splenocytes obtained from animals on day 10 post-immunization or infected animals at termination were used to assess their proliferative responses based on tritiated ^3^[H] thymidine-incorporation assay [20,21,22]. After stimulating the cells with indicated peptides for 2 days, and adding ^3^[H] thymidine (1 µci/well) during the next 16 h, proliferative responses were measured as counts per minute (cpm). In some experiments, the T cell responses are shown as fold changes derived by dividing the cpm values of cultures stimulated with peptides by the cpm values of unstimulated cultures (medium controls). Similarly, to measure proliferative responses in CD4 and CD8 T cells, lymphocytes generated from animals immunized with VP1 peptides were used to sort CD4 and CD8 T cells to a purity of more than 90% by negative selection using IMAG magnetic separation kit as recommended by the manufacturer (BD Biosciences, San Jose, CA, USA) [19].

### 2.5. MHC Class II Binding

To determine the IA^k^-binding affinities of VP1 681–700, VP1 721–740 and VP1 771–790, we used thrombin-cleaved, empty IA^k^ molecules as we have described previously [23,24,25]. Cocktails of individual reaction mixtures were prepared to include thrombin-cleaved IA^k^ monomers (0.35 µg), competitor peptides (VP1 681–700, VP1 721–740, VP1 771–790) [0.00001 µM to 100 µM], and a constant amount of biotinylated reference peptide, HEL 46–61 (1 µM) [26,27]. The mixtures were incubated at room temperature (RT) overnight. In parallel, anti-IA^k^ (10 µg/mL) was coated onto 96-well white fluorescence plates (Nunc, Rochester, NY, USA) in sodium phosphate 0.2 M, pH 6.8 overnight. After a series of washing steps, the reaction mixtures described above were added in duplicates for each peptide and incubated on a rocker at RT for 1 h and washed. Europium-labeled streptavidin (SA) was then added to a concentration of 1 µg/mL in dissociation-enhanced lanthanide fluoroimmunoassay (DELFIA) buffer followed by DELFIA-enhancement solution (Perkin Elmer, Waltham, MA, USA). After measuring the fluorescence intensities at excitation/emission wavelengths of 340/615 nm, the half-maximal inhibitory concentration (IC_50_) values were then calculated as described [20,21,22].

### 2.6. Derivation of MHC Class II Dextramers and Tetramers

We adopted two approaches to create IA^k^ dextramers and tetramers. In the first approach, we derived IA^k^ dextramers based on peptide-exchange reaction as we have described previously [25,28,29]. In brief, after biotinylation, the IA^k^/ MHC class II invariant chain-associated peptide (CLIP) precursors were treated with thrombin to release CLIP, and the peptides (VP1 681–700, VP1 721–740 and VP1 771–790) were then loaded onto empty IA^k^ molecules in peptide-exchange reactions at a 3.3 to 66.6 µM ratio. The unincorporated peptides were eliminated using Amicon ultra centrifugal filters (Millipore, Bellerica, MA, USA), and dextramers were then generated using fluorophore-conjugated streptavidin (SA). In the second approach, IA^k^ tetramers were derived by using the IA^k^ monomers in which the nucleotide (nt) sequences of VP1 681–700, VP1 721–740, VP1 771–790 and RNase 43–56 were covalently tethered into the IA^k^-β construct such that the peptide-linked monomers were expressed directly using the Baculovirus [30]. The nt sequences were: VP1 681–700 (agagatcttgaactattatacacattggtcaggcagcataaagcttacgtttatgttctg), VP1 721–740 (tcctacaaaaagggttgatgccatgcttggtactcatgtagtttgggacgtggggctaca), VP1 771–790 (gtgctggtatcaaacaa acatagtggtcccagcggatgcccaaagctcctgttacatcat) or RNase 43–56 (gtgaacacctttgtgcacgagtccctg gctgatgtccaggcc). The soluble IA^k^ molecules were then biotinylated, and tetramers were derived by using the SA-fluorophore (PJ27S, Prozyme, Hayward, CA, USA) as we have described previously [23,24].

### 2.7. Staining with Dextramers and Tetramers

LNCs and splenocytes obtained from mice immunized with VP1 681–700, VP1 721–740 and VP1 771–790 or mice infected with CVB3 or CVB4 were stimulated with the indicated peptides (20 µg/mL) for 2 days, and after obtaining the viable cells by ficoll-paque density gradient centrifugation, cells were maintained in medium containing interleukin (IL)-2. Cells were stained with dextramers or tetramers during days 8 to 9 post-stimulations followed by anti-CD4 (GK1.5, Biolegend, San Diego, CA, USA) and 7-aminoactinomycin D (7-AAD; Invitrogen, Carlsbad, CA, USA) as previously described [23,24,30]. After acquiring the cells by flow cytometry (FACSCalibur, BD Biosciences), percentages of dextramer or tetramer positive cells were determined in the live (7-AAD^¯^) CD4^+^ subset using FlowJo software (Tree Star, Ashland, OR, USA).

### 2.8. Cytokine Analysis

Supernatants were obtained from LNC and splenocyte cultures prepared from immunized animals or CVB3 and CVB4-infected animals that were stimulated with or without VP1 681–700, VP1 721–740 and VP1 771–790, and RNase 43–56 (control) (20 μg/mL) on day 3 post-stimulations. Cytokine analysis was performed using beads conjugated with capture and detection antibodies for a panel of T helper (Th) 1, (IL-2, IFN-γ), Th2 (IL-4), and Th17 (IL-17A), including other inflammatory (IL-6 and tumor necrosis factor, TNF-α) and anti-inflammatory (IL-10) cytokines as recommended by the manufacturer (BD Biosciences). Briefly, capture bead/cytokine antibody conjugates were first prepared, and the mixtures were added to a tube containing diluted standards or test samples, followed by the addition of detection antibodies. After acquiring by flow cytometry, cytokine concentrations were determined using the Flow cytometric analysis program (FCAP) Array Software (BD Biosciences).

### 2.9. Vβ Usage

LN and splenocytes were harvested from A/J mice infected with CVB3, and were stimulated with the respective peptides for 2 days (20 µg/mL) and maintained in IL-2 medium. On day 4 post-stimulation, cells were stained with a panel of anti-mouse TCR vβ antibodies: vβ 2, 4, 6, 7, 8.1, 8.2, 8.3, 9, 10b, 11, 12, 13, and 14 (BD Pharmingen), anti-CD4, and 7-AAD. After acquiring by flow cytometry, percentages of TCR vβ^+^ CD4^+^ T cells were enumerated in the live (7-AAD^¯^) population [22,31].

### 2.10. Histology

Hearts and pancreata collected at termination were fixed in 10% phosphate-buffered formalin and processed for the production of 5-µm-thick Hematoxylin and Eosin (H & E) serial sections, ~50 µm apart from each other. All sections were examined by a board-certified pathologist blinded to treatment. The total number of inflammatory cell foci was determined as reported previously [20,21].

### 2.11 Statistics

To analyze proliferative responses, baseline cpm values were derived by taking the average of the cpm values of three replicates at zero concentrations. The adjusted cpm values were then calculated as cpm values of test samples/baseline cpm values as a measure of response for test samples. Generalized linear mixed models were used to analyze the data pertaining to treatment, concentrations, and body weights. The data were analyzed using Proc Glimmix in SAS (Version 9.3, SAS Institute Inc., Cary, NC, USA). For data sets, where normality assumptions were not satisfied, Friedman’s non-parametric two-way ANOVA was used [32]. Tetramer^+^ cells were analyzed by student’s *t*-test. Barnard’s exact test was used to analyze the histological parameters [33]. The data sets shown in the figures represent the mean ± SEM values.

## 3. Results

### 3.1. CVB3 VP1 Contains Multiple Immunogenic Epitopes

**Figure 1:**
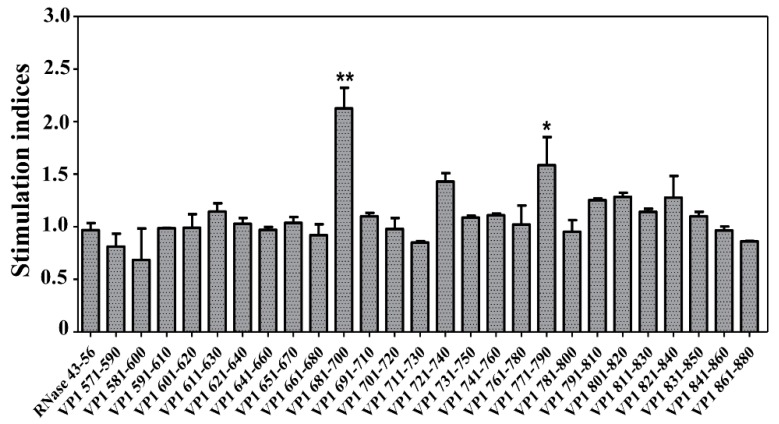
Proliferative responses to the overlapping peptides of VP1 in CVB3-infected animals. A/J mice were infected with CVB3, and at termination on day 21 post-infection, splenocytes were prepared. Cells were stimulated with or without the overlapping peptides of VP1 or control (RNase 43–56) for 2 days, and after pulsing with ^3^[H] thymidine for 16 h, proliferative responses were measured as cpm. Stimulation indices were derived by dividing the cpm values of peptide-stimulated cells (100 µg/mL) by those of medium control. Representative data set obtained from three individual experiments, each involving 10 to 12 mice are shown. * *p* ≤ 0.05, and ** *p* ≤ 0.01.

To identify the immunogenic epitopes, we focused on the VP1, since this protein has been shown to encompass antigenic determinants that induce antibody and T cell responses in other enteroviruses such as Theiler’s murine encephalomyelitis virus (TMEV) and encephalomyocarditis virus [34,35,36]. We created an overlapping peptide library consisting of 30 peptides with 10 amino acids between each (Table 1) and used them to stimulate splenocytes generated from A/J mice infected with CVB3 at 21 days post-infection based on ^3^[H] thymidine-incorporation assay. The expectation was that the VP1-sensitized T cells generated in response to CVB3 should be able to respond to some of the overlapping peptides. To capture these responses, we used RNase 43–56 as an irrelevant control. While, we noted significant responses for VP1 681–700 (*p* ≤ 0.01) and VP1 771–790 (*p* ≤ 0.05), reactivity to VP1 721–740 was marginal (Figure 1). Although similar trends were noted for few other peptides localized in the region, VP1 801–840, we did not pursue them for further characterization.

### 3.2. VP1 Epitopes Induce Mainly CD4 T Cell Responses

**Figure 2:**
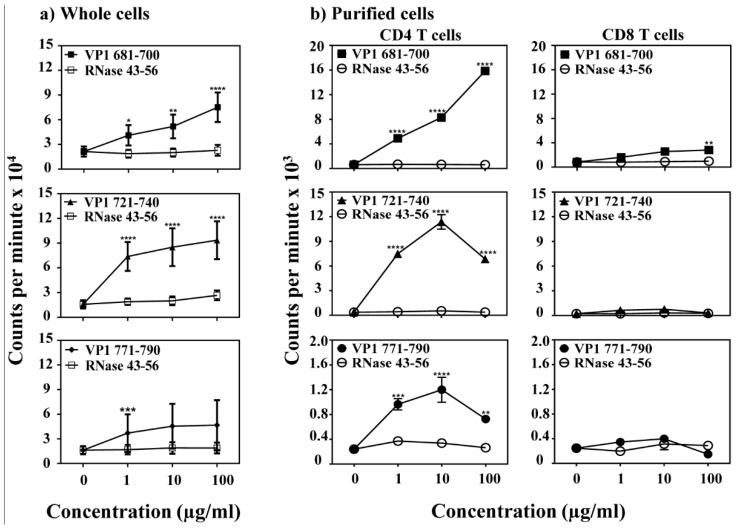
Determination of T cell responses in animals immunized with VP1 peptides. (**a**) Whole cells. LNCs and splenocytes obtained from mice immunized with VP1 681–700, VP1 721–740 and VP1 771–790 were stimulated with or without indicated VP1 peptides or control (RNase 43–56) for 2 days, and after pulsing with ^3^[H] thymidine for 16 h, proliferative responses were measured as cpm. Representative data set obtained from five individual experiments each involving three to five mice are shown. (**b**) Purified cells. LNCs obtained from mice immunized with VP1 681–700, VP1 721–740 and VP1 771–790, and CD4 and CD8 T cells were enriched using negative selection by magnetic separation. Purified cells were stimulated with the irradiated antigen presenting cells (APCs)/peptides for 2 days. After pulsing with ^3^[H] thymidine for 16 h, proliferative responses were measured as cpm. Representative data set from two individual experiments each involving three to five mice is shown. * *p* ≤ 0.05, ** *p* ≤ 0.01, *** *p* ≤ 0.001, and **** *p* ≤ 0.0001.

To characterize antigen-specificity of T cell responses induced by VP1 epitopes, we examined VP1 681–700, VP1 721–740 and VP1 771–790 by adopting an immunization strategy. LNCs obtained from immunized animals were used to analyze recall responses to the immunizing peptides by using RNase 43–56 as a control. The analysis revealed significant proliferative responses to VP1 681–700 (*p* ≤ 0.0001), VP1 721–740 (*p* ≤ 0.0001), and VP1 771–790 (*p* ≤ 0.001) (Figure 2a). Responses to all the three peptides occurred dose-dependently, and the cells did not react to control (RNase 43–56), suggesting that the responses obtained for each peptide are antigen-specific. We then asked whether the responses are restricted to CD4 or CD8 T cells using the CD4 and CD8 T cells negatively sorted by magnetic separation [19]. The data indicates that only the CD4, but not CD8 T cells, respond to each peptide (Figure 2b) except that CD8 T cells in the VP1 681–700 group responded at 100 µg/mL that might have been contributed by the residual CD4 T cells. Furthermore, by verifying the T cell responses in naïve animals, we noted no significant responses to any of the three peptides, except a low-degree of responses (~1.3-fold) were noted at a higher concentration of peptides for VP1 681–700 and VP1 721–740 (Figure S1). Thus, it is possible that the pre-existing repertoire of naïve antigen-reactive T cells can possibly expand in response to immunizations.

### 3.3. The VP1 Epitopes Bind IA^k^ Molecules Differentially and the T Cell Responses Are Antigen-Specific

**Figure 3:**
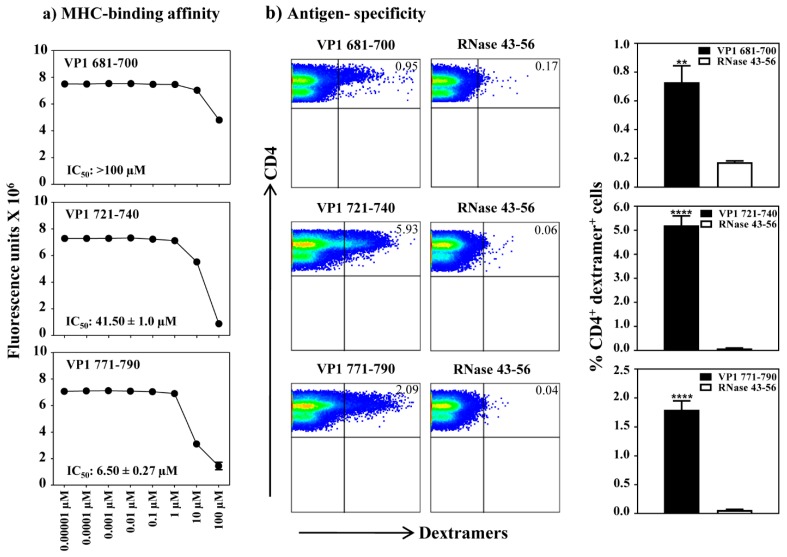
Determination of MHC-II binding affinities of VP1 epitopes and antigen-specificity of T cell responses by MHC class II dextramer staining. (**a**) MHC-binding affinity. The reaction mixtures containing thrombin-cleaved IA^k^ monomers and competitor peptides VP1 681–700, VP1 721–740 and VP1 771–790, and biotinylated reference peptide, HEL 46–61, were individually prepared and added in duplicates to fluorescence plates coated with anti-IA^k^. After washing and the addition of europium-labeled streptavidin and DELFIA-enhancer, fluorescence intensities were measured at excitation/emission wavelengths of 340/615 nm, and IC_50_ values were then calculated. Representative data from two individual experiments, each involving two replicates are shown. (**b**) Antigen-specificity. Lymphocytes prepared from animals immunized with individual VP1 peptides (VP1 681–700, VP1 721–740 or VP1 771–790) were stimulated with the corresponding peptides and were maintained in medium supplemented with IL-2 for a week. Cells were harvested between days 7–10 post-stimulation and stained with indicated IA^k^/dextramers, anti-CD4 and 7-AAD. After acquiring the cells by flow cytometry, dextramer positive cells were analyzed in the live (7-AAD¯) CD4 subset using FlowJo software. Representative flow cytometric dot-plots for specific and control dextramers are shown in the left panel. Data sets obtained from three individual experiments, each involving three to four mice are shown in the right panel. RNase 43–56, control. ** *p* ≤ 0.01, and **** *p* ≤ 0.0001.

To determine the MHC-binding affinity of VP1 epitopes, we used empty IA^k^ monomers as described previously [22]. By using HEL 46–61 as a competitor reference [26,27,37], we analyzed the binding ability of VP1 681–700, VP1 721–740 and VP1 771–790 and determined their IC_50_ values (Figure 3a) [26,27,38]. We noted that the binding affinity of VP1 771–790 (IC_50_, 6.50 ± 0.27 µM) was relatively stronger than VP1 721–740 (IC_50_, 41.50 ± 1.0 µM), whereas the IC_50_ value for VP1 681–700 was low (>100 µM). By relating these data to the T cell responses (Figure 2), it was apparent that the VP1 681–700 indeed induced T cell response in spite of being a poor IA^k^-binder. The data suggest that the poor MHC-binders do not necessarily mean that such epitopes do not induce T cell responses in vivo. We then sought to characterize antigen specificity of VP1-reactive T cells by creating MHC class II/IA^k^ dextramers for all the three epitopes. Essentially, we generated empty IA^k^ monomers as we previously described [23,24,29], and after biotinylation, the IA^k^ molecules were loaded with VP1 681–700, VP1 721–740 and VP1 771–790 in a peptide-exchange reaction, which were then used to generate dextramers after conjugating with SA-fluorophores. By using these reagents and control dextramers (RNase 43–56), we performed the dextramer staining using T cell cultures generated for each peptide. While, CD4 T cells in all the three cultures were found to bind the corresponding dextramers, but not control, the dextramer staining intensity occurred in the order of VP1 721–740 (5.93%) followed by VP1 771–790 (2.09%) and VP1 681–700 (0.95%) (Figure 3b). The data pointed to a possibility that the VP1 epitopes may bind MHC class II molecules differentially.

### 3.4. CVB3 Infection Leads to the Induction of VP1 Virus-Specific, CD4 T Cell Responses

**Figure 4:**
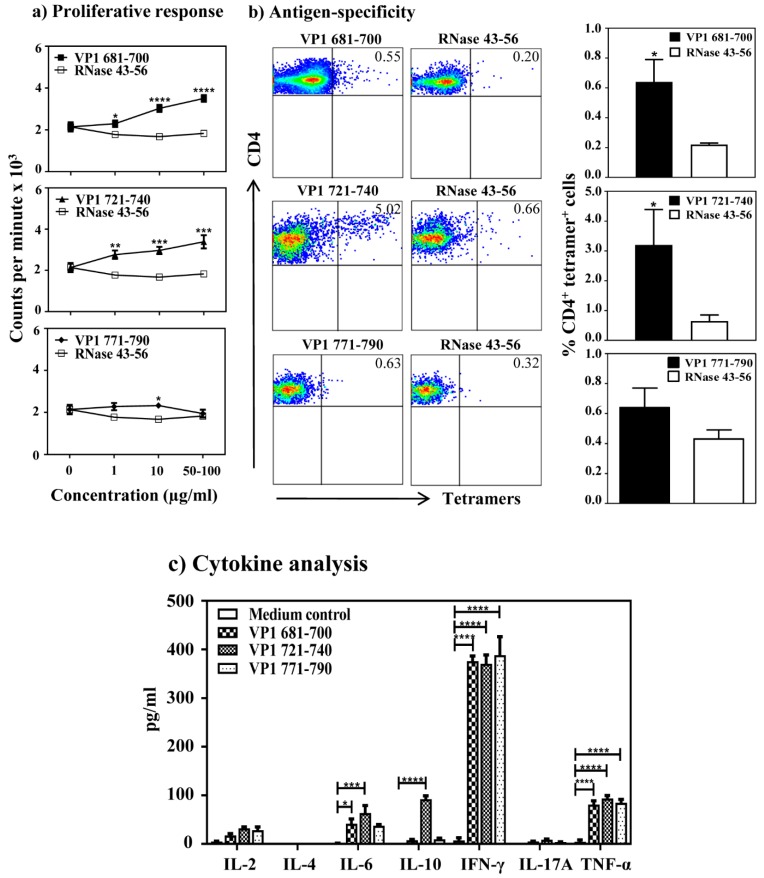
Evaluation of virus-specific T cell responses in infected animals. (**a**) Proliferative response. A/J mice were infected with CVB3, and at termination on day 21 post-infection, splenocytes were stimulated with or without VP1 681–700, VP1 721–740 and VP1 771–790 or control (RNase 43–56) for 2 days, and after pulsing with ^3^[H] thymidine for 16 h, proliferative responses were measured as cpm. Data sets obtained from three individual experiments each involving six to eight mice are shown. (**b**) Antigen-specificity. Splenocytes from CVB3-infected mice were stimulated with indicated VP1 peptides and maintained in medium containing IL-2 for a week. Viable cells were harvested between days 7–10 post-stimulation and stained with indicated IA^k^/tetramers, anti-CD4 and 7-AAD. After acquiring the cells by flow cytometry, tetramer positive cells were analyzed in the live (7-AAD¯) CD4 subset using FlowJo software. Representative flow cytometric dot-plots for specific and control tetramers are shown in the left panel. Data sets obtained from three individual experiments, each involving six to eight mice are indicated in the right panel. RNase 43–56, control. (**c**) Cytokine analysis. Splenocytes from the CVB3-infected mice were stimulated with or without the indicated peptides. Supernatants collected on day 3 post-stimulation were analyzed for indicated cytokines by cytokine bead array. Data from three individual experiments, each involving six to eight mice, are shown. * *p* ≤ 0.05, ** *p* ≤ 0.01, *** *p* ≤ 0.001, and **** *p* ≤ 0.0001.

The availability of three VP1 epitopes that induce varied T cell responses offered an advantage to investigate the appearance of virus-specific T cells in CVB3 infection with an expectation that the T cell reactivity can be obtained for at least one or more of these epitopes. We tested this hypothesis by proliferation assay using lymphocytes generated from CVB3-infected animals that were later stimulated with individual VP1 peptides (VP1 681–700, VP1 721–740 and VP1 771–790). We noted the T cell responses to be dose-dependent, and significant for VP1 681–700 (*p* ≤ 0.0001) and VP1 721–740 (*p* ≤ 0.0001), but not for VP1 771–790 (Figure 4a). Expectedly, lymphocytes were non-responsive to control (RNase 43–56). Additionally, based on TCR vβ analysis, we noted no striking differences with the use of specific vβ’s between any of the three peptide stimulations, and vβ-usage occurred in the order of vβ8.1, 8.2 followed by others (vβ4, 6, 8.3, 10b, 14, 7, 2, 13, 12 and 9) (Figure S2). We then determined antigen-specificity by creating the MHC class II tetramers (IA^k^), since the availability of dextramers became limited. We also decided to generate fresh IA^k^ monomers based on peptide-tethered strategy, where the nucleotide sequences for VP1 681–700, VP1 721–740 and VP1 771–790 were tethered into the IA^k^-β constructs as described previously [23,24,39]. After validating the tetramer reagents in the immunization settings [22,23], we used them to stain lymphocytes prepared from CVB3-infected mice (Figure 4b). While, the CD4 T cells reactive with VP1 681–700 and VP1 721–740 were found to bind to their corresponding tetramers with the staining intensity for VP1 721–740 (3.20 ± 1.19 %, *p* < 0.05) to be relatively higher than VP1 681–700 (0.64 ± 0.15 %, *p* < 0.05) tetramers, no appreciable tetramer staining was evident for VP1 771–790 (0.64 ± 0.13 %) tetramers when compared with the control tetramers (RNase 43–56). These data complement the patterns obtained in the proliferation assay described above.

We then measured cytokine production in the culture supernatants obtained from lymphocyte cultures stimulated with or without VP1 681–700, VP1 721–740 and VP1 771–790 by bead array analysis for a panel of Th1, Th2, Th17 and other inflammatory (IL-6 and TNF-α) and anti-inflammatory (IL-10) cytokines. While we detected the secretion of all cytokines tested except IL-4, their patterns varied (Figure 4c). (a) The predominant cytokine observed in all the three peptide-stimulations was IFN-γ (371 ± 18 to 388 ± 38 pg/mL). (b) In contrast to VP1 681–700 and VP1 771–790, we noted the detection of other cytokines in VP1 721–740-stimulated cultures in the order of TNF-α (93 ± 6 pg/mL), IL-10 (92 ± 7 pg/mL), IL-6 (64 ± 15 pg/mL), IL-2 (32 ± 3 pg/mL), and IL-17A (9 ± 1 pg/mL). Since IFN-γ is considered as a prototypic Th1 cytokine, and all the three peptides induced IFN-γ, we propose that the IFN-γ-producing virus-reactive T cells may have a role in the protection against CVB3 infection.

### 3.5. Multiple Serotypes of CVB Can Potentially Induce Similar T Cell Responses

**Figure 5:**
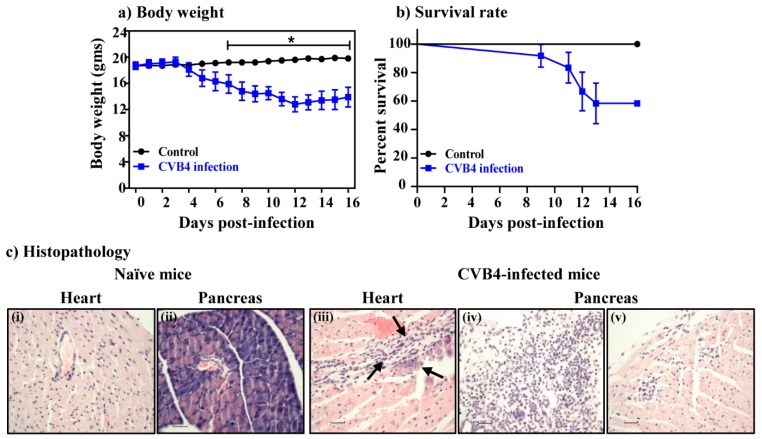
Evaluation of disease severity induced with CVB4 in A/J mice. (**a**) Body weight. A/J mice were infected with or without CVB4 and their body weights were noted up to 16 days post-infection. (**b**) Survival rates. The above mice were monitored for mortality post-infection and their survival rates were calculated. Data from two individual experiments, each involving four to eight mice are shown. (**c**) Histopathology. Hearts and pancreata collected at termination on day 16 post-infection with CVB4 were evaluated for inflammatory changes. While, heart (i) and pancreatic (ii) sections from control group were normal, sections from CVB4-infected mice had hemorrhagic necrosis, fibrosis, mineralization and inflammation in heart (iii), and atrophy (iv) and inflammation (v) in the pancreas. Original magnification, ×40. Representative data from the above experiments are shown. * *p* ≤ 0.05.

To determine whether the T cell epitopes identified within the CVB3 VP1 can also be used to evaluate T cell responses in other serotypes, we compared the amino acid sequences of VP1 681–700, VP1 721–740 and VP1 771–790 between the six serotypes of CVB. These analyses revealed identities with a range of 65% to 85% for VP1 681–700, 75% to 90% for VP1 721–740 and 75% to 95% for VP1 771–790 (Figure S3, left panel). We also localized these epitopes in the VP1 capsid protomers within the viral canyon by using the three dimensional structure of CVB3 (Figure S3, top and bottom right panel) [40]. The data imply that some of these epitopes can potentially interact with the host receptor, coxsackievirus-adenovirus receptor, leading to the induction of immune response, if immune cells are targeted. Since we had access to CVB4, the serotype implicated in the T1D [41], we asked whether the CVB4 infection can also lead to the induction of VP1-specific T cell responses. First, we established an infection model in A/J mice, since CVB4 infection has not been tested in this mouse strain, and a viral dose of 2,000 TCID_50_ was found to induce mainly pancreatitis and mild myocarditis in this mouse strain. Clinically, the infected animals showed progressive loss of body weight beginning from day 3 and continued for up to day 16 post-infection (Figure 5a), whereas mortalities were noted in 42% of animals (Figure 5b). Histologically, heart and pancreatic sections from naïve animals did not reveal any lesions as expected. However, 33% of animals infected with CVB4 had mild myocarditis, and heart sections revealed infiltrations of mononuclear cells (MNCs) and histiocytes (Figure 5c and Table 2, top panel). In contrast, 92% of CVB4-infected animals showed pancreatic involvement, and acinar atrophy and inflammation were detected in 83% and 50% of animals as prominent features, whereas necrosis was noted in 17% of animals (Figure 5c and Table 2, bottom panel).

**Figure 6:**
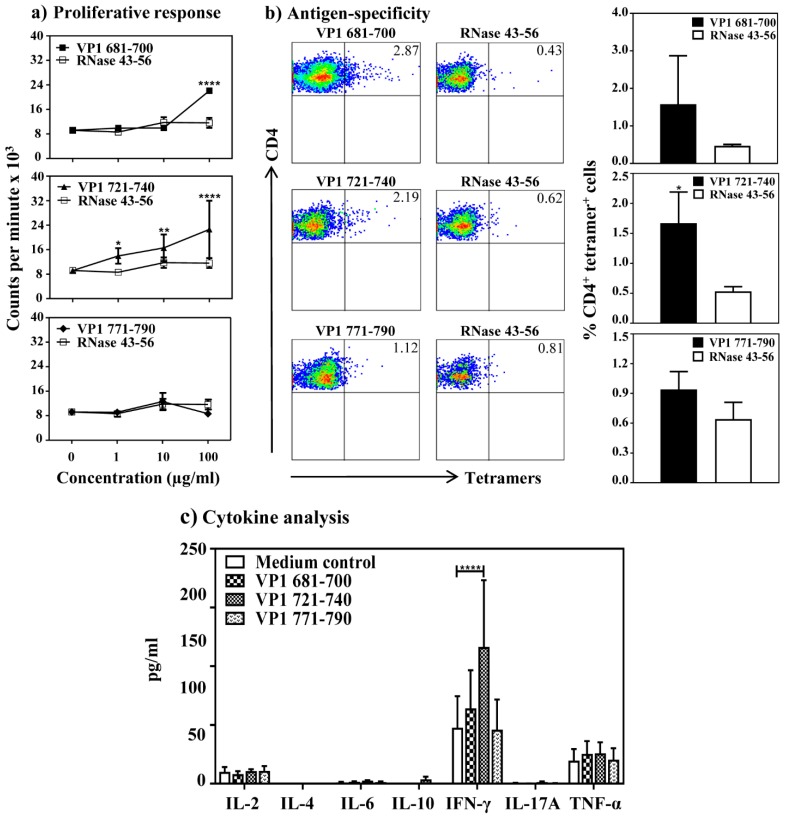
Evaluation of antigen-specific T cell responses in CVB4 infected mice: (**a**) Proliferative response. A/J mice were infected with CVB4, and at termination, splenocytes were stimulated with or without VP1 681–700, VP1 721–740 and VP1 771–790 or control (RNase 43–56) for 2 days, and after pulsing with ^3^[H] thymidine for 16 h, proliferative responses were measured as cpm. Representative data set obtained from two individual experiments each involving four to eight mice are shown. (**b**) Antigen-specificity. Splenocytes from CVB4-infected mice were stimulated with indicated peptides and maintained in medium containing IL-2 for a week. Cells harvested between days 7–10 post-stimulation were stained with the indicated IA^k^/tetramers, anti-CD4 and 7-AAD. After acquiring the cells by flow cytometry, tetramer positive cells were analyzed in the live (7-AAD¯) CD4 subset using FlowJo software. Representative flow cytometric dot-plots for specific and control tetramers are shown in the left panel. Data sets obtained from three individual experiments, each involving four to eight mice are indicated in the right panel for each tetramer reagent. RNase 43–56, control. (**c**) Cytokine analysis. Splenocytes from the CVB4-infected mice were stimulated with or without the indicated peptides. Supernatants were collected on day 3 post-stimulation and were analyzed for cytokines by cytokine bead array. Data from three individual experiments each involving four to eight mice are shown. * *p* ≤ 0.05, ** *p* ≤ 0.01, and **** *p* ≤ 0.0001.

We then asked whether CVB4 infection can lead to VP1-specific T cell responses similar to CVB3 infection. This was investigated by harvesting the splenocytes from CVB4-infected animals, and evaluated their response to VP1 681–700, VP1 721–740 or VP1 771–790 based on ^3^[H] thymidine-incorporation assay. The data indicate that the T cell responses were noted predominantly for VP1 721–740 (*p* ≤ 0.01), whereas no significant responses were noted for VP1 681–700 or VP1 771–790 (Figure 6a). The responses were specific as the cells did not respond to control (RNase 43–56). To further evaluate their antigen-specificity, we used MHC class II (IA^k^) tetramers for VP1 681–700, VP1 721–740 or VP1 771–790 and stained the lymphocytes stimulated with the corresponding peptides. The flow cytometric analysis revealed staining for VP1 681–700 and VP1 721–740 tetramers, and their respective staining intensities were 2.9% and 2.2% with an increase of ~4 to 7-fold over control tetramers (RNase 43–56) (Figure 6b). Expectedly, however, the staining obtained with VP1 771–790 tetramers was low over control (~1.3-fold). Additionally, by evaluating cytokine responses, we found that the culture supernatants obtained from VP1 721–740-stimulation had IFN-γ (116 ± 57 pg/mL). A similar trend was noted with VP1 681–700, but not for VP1 771–790 (Figure 6c). Conversely, none of the other cytokines (IL-2, IL-6, IL-10, IL-17A and TNF-α) were found elevated in any of the culture supernatants as compared to medium control. However, IL-4 secretion was lacking in all of them. Taken together, the data suggest that the anti-viral T cell responses in CVB4 infection can be captured with the assays developed for VP1 721–740, and the next best candidate is VP1 681–700.

## 4. Discussion

In this study, we describe the identification of three T cell epitopes within the CVB3 VP1 that can be used to evaluate antigen-specific T cell responses in CVB infections caused by multiple serotypes. CVB3 VP1 has been shown to contain several immunogenic epitopes [42], but it was unknown whether active infection with CVB3 can lead to the generation of virus-reactive T cell responses. Similarly, their antigen-specificity remained uninvestigated. Furthermore, it is well known that multiple serotypes of CVB such as CVB3 and CVB4 can induce similar diseases such as myocarditis and pancreatitis [43,44,45,46]. Therefore, development of tools common to multiple serotypes may be helpful to analyze immune responses in such infections. To this end, we made an effort to identify the T cell epitopes with an intent to evaluate antigen-specific T cell responses in infections caused by CVB3 and CVB4, since these serotypes are commonly implicated in the causation of DCM and T1D, respectively [5,41].

Our investigations involved the use of A/J mice that are susceptible for CVB3 infection. To identify the T cell epitopes, we created the overlapping peptide library consisting of 30 peptides, and their ability to stimulate splenocytes from CVB3 infected animals. This approach was helpful since we could narrow down to three peptides (VP1 681–700, VP1 721–740 and VP1 771–790) as potential candidates by eliminating 27 other peptides as non-stimulators of T cell responses. By reasoning that these epitopes could be naturally presented by the APCs in vivo, we chose them for further characterization.

Furthermore, we ascertained that CD4 T cells, but not CD8 T cells, respond to VP1 epitopes with a tendency for proliferative responses to be more for VP1 681–700 and VP1 721–740 followed by VP1 771–790. Additionally, by using MHC class II dextramers, we verified that T cell responses induced by each peptide were also antigen-specific. However, the MHC class II (IA^k^)-binding assay revealed that VP1 681–700 was found to be a poor binder, yet it induced good T cell responses. A/J mice express two MHC class II alleles namely, IA^k^ and IE^k^ [23,47], and it is possible that VP1 681–700 may bind IE^k^ molecule, which we have not investigated here. Alternatively, it is possible that the autoantigens that bind poorly to MHC molecules can still elicit immune response as reported for myelin basic protein [48]. On the other hand, VP1 771–790 in spite of being a good MHC-binder, induced relatively moderate proliferative response. Thus, our data suggest that variations in the MHC-binding affinity of T cell epitopes intramolecularly, as in the case of VP1, may or may not influence the T cell responses. In such instances, determination of T cell responses for multiple epitopes may be helpful. Since all the three VP1 epitopes induced T cell responses in the immunization settings, we sought to enumerate the frequencies of antigen-specific CD4 T cells in CVB3 infection by creating IA^k^ tetramers for VP1 681–700, VP1 721–740 and VP1 771–790. We noted detection of CD4 T cells specific to VP1 721–740 consistently, more than VP1 681–700, but VP1 771–790-specific CD4 T cells were low as evaluated by MHC tetramer staining. Nonetheless, all three epitopes induced IFN-γ responses. Furthermore, production of IFN-γ as in the case of VP1 771–790, but the absence of significant expansion of antigen-specific T cells, may mean that cytokines might have been produced from non-antigen-specific T cells through, potentially, bystander activation [49]. Thus, our data support a notion that evaluation of antigen-specific T cell responses by different assays can yield data that may or may not complement with each other.

Finally, we expanded our observations to ask a question whether the VP1 epitopes that we have identified can be used to evaluate T cell responses in other serotypes of CVB. We addressed this question by establishing CVB4 infection model in A/J mice, in which the virus was found to induce mainly pancreatitis, whereas myocarditis was mild. In this setting, we noted the virus-reactive T cell responses to be dominant mainly for VP1 721–740 and VP1 681–700. However, by relating the cytokine production, particularly IFN-γ, with MHC class II tetramer staining analysis, it was apparent that VP1 721–740-reactivity can be considered as a reliable candidate. Previous reports provide evidence for the occurrence of T cell responses to structural (VP1 to VP3) and non-structural (P2C) proteins of CVB4 in the T1D patients, suggesting the existence of T cell epitopes in these proteins [50]. Therefore, it is possible that other VPs (VP2 and VP3) may also encompass T cell epitopes, which we have not investigated here. Nonetheless, if the objective is to investigate antigen-specific, anti-viral T cell responses experimentally, then VP1 721–740 can be used as a primary candidate.

In summary, we have identified three T cell epitopes, and at least one epitope, VP1 721–740, can be used to evaluate antigen-specific, T cell responses both by proliferation assay and also by tetramer staining at a single cell level by flow cytometry in CVB infections caused by more than one serotype. Here we have provided evidence for two serotypes namely CVB3 and CVB4, and because both CVB3 and CVB4 viruses have been implicated in the causation, respectively, in viral myocarditis/DCM and T1D, and both infections induce similar T cell responses, we propose that the VP1 721–740 and VP1 681–700 specific MHC class II/IA^k^ tetramers can be used as helpful tools to analyze the frequencies of antigen-specific T cells in CVB infection or vaccine studies involving these serotypes. Translationally, whether these epitopes can be used to analyze T cell reactivity in human settings needs detailed analysis. Nevertheless, we performed bioinformatics analysis using immune epitope database (IEDB) [51], leading us to identify 3 to 10 putative epitopes within VP1 681–700, VP1 721–740 or VP1 771–790 that can potentially bind several HLA class I and class II alleles (Table S1). Since humans are the natural hosts for CVB infections [52], and mouse models are commonly employed to understand the disease pathogenesis and antiviral immune mechanisms of these infections, the availability of our tools described in this study may be helpful to evaluate T cell responses in various experimental models.

## Figures and Tables

**Table 1:** List of CVB3 VP1 overlapping peptides

Peptide	Sequence	Peptide	Sequence
VP1 571–590	GPVEDAITAAIGRVADTVGT	VP1 721–740	PDKVDSYVWQTSTNPSVFWT
VP1 581–600	IGRVADTVGTGPNNSEAIPA	VP1 731–750	TSTNPSVFWTEGNAPPRMSI
VP1 591–610	GPNNSEAIPALTAAETGHTS	VP1 741–760	EGNAPPRMSIPFLSIGNAYS
VP1 601–620	LTAAETGHTSQVVPGDTMQT	VP1 751–770	PFLSIGNAYSNFYDGWSEFS
VP1 611–630	QVVPGDTMQTRHVKNYHSRS	VP1 761–780	NFYDGWSEFSRNGVYGINTL
VP1 621–640	RHVKNYHSRSESTIENFLCR	VP1 771–790	RNGVYGINTLNNMGTLYARH
VP1 631–650	ESTIENFLCRSACVYFTEYE	VP1 781–800	NNMGTLYARHVNAGSTGPIK
VP1 641–660	SACVYFTEYENSGAKRYAEW	VP1 791–810	VNAGSTGPIKSTIRIYFKPK
VP1 651–670	NSGAKRYAEWVLTPRQAAQL	VP1 801–820	STIRIYFKPKHVKAWIPRPP
VP1 661–680	VLTPRQAAQLRRKLEFFTYV	VP1 811–830	HVKAWIPRPPRLCQYEKAKN
VP1 671–690	RRKLEFFTYVRFDLELTFVI	VP1 821–840	RLCQYEKAKNVNFQPSGVTT
VP1 681–700	RFDLELTFVITSTQQPSTTQ	VP1 831–850	VNFQPSGVTTTRQSITTMTN
VP1 691–710	TSTQQPSTTQNQDAQILTHQ	VP1 841–860	TRQSITTMTNTGAIWTTIRG
VP1 701–720	NQDAQILTHQIMYVPPGGPV	VP1 851–870	TGAIWTTIRGSVCGDYRVVN
VP1 711–730	IMYVPPGGPVPDKVDSYVWQ	VP1 861–880	SVCGDYRVVNRHSATSADWQ

**Table 2:** Histological evaluation of hearts and pancreata in naïve and CVB4-infected mice.

Parameters	Naïve	CVB4
Heart		
Incidence	0/4 (0.0)	4/12 (33.3)
Mortality	0/4 (0.0)	5/12 (41.7)
Myocardial lesions	0.0 (0.0)	10.0 ± 1.2
Pancreas		
Incidence	0 (0.0)	11/12 (91.7)
Atrophy	0 (0.0)	10/12 (83.3)
Inflammation	0 (0.0)	6/12 (50.0)
Necrosis	0 (0.0)	2/12 (16.7)
Mineralization	0 (0.0)	0/12 (0.0)

() indicates percentages.

## Supplementary Materials

The following are available online at https://www.mdpi.com/1999-4915/12/3/347/s1.

## Abbreviations

DCM	Dilated cardiomyopathy
CVB	Coxsackievirus B
T1D	Type 1 diabetes
MHC	major histocompatibility complex
CPE	cytopathic effect
RNase	bovine ribonuclease
HEL	hen egg lysozyme
APCs	antigen presenting cells
PBS	phosphate-buffered saline
LNCs	lymph node cells
EMEM	Eagle’s minimum essential medium
FBS	fetal bovine serum
^3^[H]	tritiated-thymidine
Cpm	counts per minute
HLA	human leukocyte antigen
IEDB	immune epitope database
IL	Interleukin
7-AAD	7-aminoactinomycin-D
IFN	interferon
VP	viral protein
TMEV	Theiler’s murine encephalomyelitis virus
DELFIA	dissociation-enhanced lanthanide fluoro immuno assay
Th	T helper
CLIP	class II invariant chain-associated peptide
TNF	tumor necrosis factor
MBP	myelin basic protein
SA	streptavidin
CFA	Complete Freund’s adjuvant

## References

[1] Zaoutis T., Klein J.D. (1998). Enterovirus infections. Pediatr. Rev. Am. Acad. Pediatr..

[2] Rhoades R.E., Tabor-Godwin J.M., Tsueng G., Feuer R. (2011). Enterovirus infections of the central nervous system. Virology.

[3] Tracy S., Hofling K., Pirruccello S., Lane P.H., Reyna S.M., Gauntt C.J. (2000). Group B coxsackievirus myocarditis and pancreatitis: Connection between viral virulence phenotypes in mice. J. Med. Virol..

[4] Archard L.C., Bowles N.E., Cunningham L., Freeke C.A., Olsen E.G., Rose M.L., Meany B., Why H.J., Richardson P.J. (1991). Molecular probes for detection of persisting enterovirus infection of human heart and their prognostic value. Eur. Heart J..

[5] Cihakova D., Rose N.R. (2008). Pathogenesis of myocarditis and dilated cardiomyopathy. Adv. Immunol..

[6] Kuhl U., Pauschinger M., Noutsias M., Seeberg B., Bock T., Lassner D., Poller W., Kandolf R., Schultheiss H.P. (2005). High prevalence of viral genomes and multiple viral infections in the myocardium of adults with “idiopathic” left ventricular dysfunction. Circulation.

[7] Martino T., Liu P., Sole M.J. (1995). Enteroviral myocarditis and dialted cardiomyopathy: A review of clinical and experimental studies. Human Enterovirus Infections.

[8] Chapman N.M., Kim K.S. (2008). Persistent coxsackievirus infection: Enterovirus persistence in chronic myocarditis and dilated cardiomyopathy. Curr. Top. Microbiol. Immunol..

[9] Rose N.R., Wolfgram L.J., Herskowitz A., Beisel K.W. (1986). Postinfectious autoimmunity: Two distinct phases of coxsackievirus B3-induced myocarditis. Ann. N. Y. Acad. Sci..

[10] Yoon J.W., Austin M., Onodera T., Notkins A.L. (1979). Isolation of a virus from the pancreas of a child with diabetic ketoacidosis. N. Engl. J. Med..

[11] Dotta F., Censini S., van Halteren A.G., Marselli L., Masini M., Dionisi S., Mosca F., Boggi U., Muda A.O., Del Prato S. (2007). Coxsackie B4 virus infection of beta cells and natural killer cell insulitis in recent-onset type 1 diabetic patients. Proc. Natl. Acad. Sci. USA.

[12] Clements G.B., Galbraith D.N., Taylor K.W. (1995). Coxsackie B virus infection and onset of childhood diabetes. Lancet.

[13] Gamble D.R., Kinsley M.L., FitzGerald M.G., Bolton R., Taylor K.W. (1969). Viral antibodies in diabetes mellitus. Br. Med. J..

[14] Drescher K.M., Tracy S.M. (2008). The CVB and etiology of type 1 diabetes. Curr. Top. Microbiol. Immunol..

[15] Tracy S., Drescher K.M. (2007). Coxsackievirus infections and NOD mice: Relevant models of protection from, and induction of, type 1 diabetes. Ann. N. Y. Acad. Sci..

[16] Tracy S., Drescher K.M., Chapman N.M., Kim K.S., Carson S.D., Pirruccello S., Lane P.H., Romero J.R., Leser J.S. (2002). Toward testing the hypothesis that group B coxsackieviruses (CVB) trigger insulin-dependent diabetes: Inoculating nonobese diabetic mice with CVB markedly lowers diabetes incidence. J. Virol..

[17] Filippi C.M., Estes E.A., Oldham J.E., von Herrath M.G. (2009). Immunoregulatory mechanisms triggered by viral infections protect from type 1 diabetes in mice. J. Clin. Investig..

[18] Cihakova D., Barin J.G., Afanasyeva M., Kimura M., Fairweather D., Berg M., Talor M.V., Baldeviano G.C., Frisancho S., Gabrielson K. (2008). Interleukin-13 protects against experimental autoimmune myocarditis by regulating macrophage differentiation. Am. J. Pathol..

[19] Massilamany C., Gangaplara A., Basavalingappa R.H., Rajasekaran R.A., Khalilzad-Sharghi V., Han Z., Othman S., Steffen D., Reddy J. (2016). Localization of CD8 T cell epitope within cardiac myosin heavy chain-α 334–352 that induces autoimmune myocarditis in A/J mice. Int. J. Cardiol..

[20] Basavalingappa R.H., Massilamany C., Krishnan B., Gangaplara A., Kang G., Khalilzad-Sharghi V., Han Z., Othman S., Li Q., Riethoven J.-J. (2016). Identification of an Epitope from Adenine Nucleotide Translocator 1 That Induces Inflammation in Heart in A/J Mice. Am. J. Pathol..

[21] Krishnan B., Massilamany C., Basavalingappa R.H., Gangaplara A., Kang G., Li Q., Uzal F.A., Strande J.L., Delhon G.A., Riethoven J.J. (2017). Branched chain α-ketoacid dehydrogenase kinase 111–130, a T cell epitope that induces both autoimmune myocarditis and hepatitis in A/J mice. Immun. Inflamm. Dis..

[22] Massilamany C., Gangaplara A., Steffen D., Reddy J. (2011). Identification of novel mimicry epitopes for cardiac myosin heavy chain-α that induce autoimmune myocarditis in A/J mice. Cell. Immunol..

[23] Massilamany C., Gangaplara A., Chapman N., Rose N., Reddy J. (2011). Detection of cardiac myosin heavy chain-alpha-specific CD4 cells by using MHC class II/IA(k) tetramers in A/J mice. J. Immunol. Methods.

[24] Reddy J., Bettelli E., Nicholson L., Waldner H., Jang M.H., Wucherpfennig K.W., Kuchroo V.K. (2003). Detection of autoreactive myelin proteolipid protein 139-151-specific T cells by using MHC II (IAs) tetramers. J. Immunol..

[25] Day C.L., Seth N.P., Lucas M., Appel H., Gauthier L., Lauer G.M., Robbins G.K., Szczepiorkowski Z.M., Casson D.R., Chung R.T. (2003). Ex vivo analysis of human memory CD4 T cells specific for hepatitis C virus using MHC class II tetramers. J. Clin. Investig..

[26] Hausmann D.H., Yu B., Hausmann S., Wucherpfennig K.W. (1999). pH-dependent peptide binding properties of the type I diabetes-associated I-Ag7 molecule: Rapid release of CLIP at an endosomal pH. J. Exp. Med..

[27] Fugger L., Liang J., Gautam A., Rothbard J.B., McDevitt H.O. (1996). Quantitative analysis of peptides from myelin basic protein binding to the MHC class II protein, I-Au, which confers susceptibility to experimental allergic encephalomyelitis. Mol. Med..

[28] Jang M.H., Seth N.P., Wucherpfennig K.W. (2003). Ex vivo analysis of thymic CD4 T cells in nonobese diabetic mice with tetramers generated from I-A(g7)/class II-associated invariant chain peptide precursors. J. Immunol..

[29] Massilamany C., Upadhyaya B., Gangaplara A., Kuszynski C., Reddy J. (2011). Detection of autoreactive CD4 T cells using major histocompatibility complex class II dextramers. BMC Immunol..

[30] Massilamany C., Steffen D., Reddy J. (2010). An epitope from Acanthamoeba castellanii that cross-react with proteolipid protein 139-151-reactive T cells induces autoimmune encephalomyelitis in SJL mice. J. Neuroimmunol..

[31] Massilamany C., Thulasingam S., Steffen D., Reddy J. (2011). Gender differences in CNS autoimmunity induced by mimicry epitope for PLP 139-151 in SJL mice. J. Neuroimmunol..

[32] Daniel W.W. (1978). Applied Nonparametric Statistics.

[33] Barnard G.A. (1945). A New Test for 2 × 2 Tables. Nature.

[34] Yauch R.L., Kim B.S. (1994). A predominant viral epitope recognized by T cells from the periphery and demyelinating lesions of SJL/J mice infected with Theiler’s virus is located within VP1(233-244). J. Immunol..

[35] Zurbriggen A., Hogle J.M., Fujinami R.S. (1989). Alteration of amino acid 101 within capsid protein VP-1 changes the pathogenicity of Theiler’s murine encephalomyelitis virus. J. Exp. Med..

[36] Walker E.J., Jeffrey P.D. (1988). Sequence homology between encephalomyocarditis virus protein VPI and histidyl-tRNA synthetase supports a hypothesis of molecular mimicry in polymyositis. Med. Hypotheses.

[37] Babbitt B.P., Allen P.M., Matsueda G., Haber E., Unanue E.R. (1985). Binding of immunogenic peptides to Ia histocompatibility molecules. Nature.

[38] Basavalingappa R.H., Massilamany C., Krishnan B., Gangaplara A., Rajasekaran R.A., Afzal M.Z., Riethoven J.J., Strande J.L., Steffen D., Reddy J. (2017). beta1-Adrenergic Receptor Contains Multiple IA(k) and IE(k) Binding Epitopes That Induce T Cell Responses with Varying Degrees of Autoimmune Myocarditis in A/J Mice. Front. Immunol..

[39] Massilamany C., Gangaplara A., Jia T., Elowsky C., Kang G., Riethoven J.-J., Li Q., Zhou Y., Reddy J. (2014). Direct staining with major histocompatibility complex class II dextramers permits detection of antigen-specific, autoreactive CD4 T cells in situ. PLoS ONE.

[40] Muckelbauer J.K., Kremer M., Minor I., Tong L., Zlotnick A., Johnson J.E., Rossmann M.G. (1995). Structure determination of coxsackievirus B3 to 3.5 A resolution. Acta Cryst. D Biol. Cryst..

[41] Jaidane H., Hober D. (2008). Role of coxsackievirus B4 in the pathogenesis of type 1 diabetes. Diabetes Metab..

[42] Huber S., Polgar J., Moraska A., Cunningham M., Schwimmbeck P., Schultheiss P. (1993). T lymphocyte responses in CVB3-induced murine myocarditis. Scand. J. Infect. Dis. Suppl..

[43] Blay R., Simpson K., Leslie K., Huber S. (1989). Coxsackievirus-induced disease. CD4+ cells initiate both myocarditis and pancreatitis in DBA/2 mice. Am. J. Pathol..

[44] Vella C., Brown C.L., McCarthy D.A. (1992). Coxsackievirus B4 infection of the mouse pancreas: Acute and persistent infection. J. Gen. Virol..

[45] Baxter N.J., Roetzer A., Liebig H.D., Sedelnikova S.E., Hounslow A.M., Skern T., Waltho J.P. (2006). Structure and dynamics of coxsackievirus B4 2A proteinase, an enyzme involved in the etiology of heart disease. J. Virol..

[46] Fairweather D., Stafford K.A., Sung Y.K. (2012). Update on coxsackievirus B3 myocarditis. Curr. Opin. Rheumatol..

[47] Hirayama M., Azuma E., Jiang Q., Kobayashi M., Iwamoto S., Kumamoto T., Kisenge R., Yamamoto H., Komada Y. (2000). The reconstitution of CD45RBhiCD4+ naive T cells is inversely correlated with donor age in murine allogeneic haematopoietic stem cell transplantation. Br. J. Haematol..

[48] Lee C., Liang M.N., Tate K.M., Rabinowitz J.D., Beeson C., Jones P.P., McConnell H.M. (1998). Evidence that the autoimmune antigen myelin basic protein (MBP) Ac1-9 binds towards one end of the major histocompatibility complex (MHC) cleft. J. Exp. Med..

[49] Boyman O. (2010). Bystander activation of CD4+ T cells. Eur. J. Immunol..

[50] Varela-Calvino R., Ellis R., Sgarbi G., Dayan C.M., Peakman M. (2002). Characterization of the T-cell response to coxsackievirus B4: Evidence that effector memory cells predominate in patients with type 1 diabetes. Diabetes.

[51] Vita R., Mahajan S., Overton J.A., Dhanda S.K., Martini S., Cantrell J.R., Wheeler D.K., Sette A., Peters B. (2019). The Immune Epitope Database (IEDB): 2018 update. Nucleic Acids Res..

[52] Sin J., Mangale V., Thienphrapa W., Gottlieb R.A., Feuer R. (2015). Recent progress in understanding coxsackievirus replication, dissemination, and pathogenesis. Virology.

