# Supplementary material for: Identification of Immunogenic Epitopes That Permit the Detection of Antigen-Specific T Cell Responses in Multiple Serotypes of Group B Coxsackievirus Infections
Source: Viruses. 2020 Mar 21;12(3):347. doi: 10.3390/v12030347 (PMC7150766; doi:10.3390/v12030347)
Supplement: Supplementary file 1 [file viruses-12-00347-s001.zip › Supplementary/Supplementary Table 1.docx]

| **Table S1: Potential VP1 T cell epitopes binding to HLA alleles in humans** | | | | |
| --- | --- | --- | --- | --- |
| Epitope | Sequence | HLA allele | Length | Percentile |
| MHC class I Alleles | | | | |
| VP1 681-700 | RFDLELTFV | HLA-C*04:01 | 9 | 0.03 |
|  | FDLELTFVI | HLA-B*13:02 | 9 | 0.08 |
|  | FDLELTFVI | HLA-B*52:01 | 9 | 0.12 |
|  | LELTFVITS | HLA-B*50:01 | 9 | 0.21 |
|  | RFDLELTFV  FDLELTFVI  RFDLELTFV  FDLELTFVI  RFDLELTFV  LELTFVITS | HLA-C*05:01  HLA-B*37:01  HLA-C*07:04  HLA-B*49:01  HLA-G*01:01  HLA-B*18:01 | 9  9  9  9  9  9 | 0.42  0.42  0.48  0.66  0.71  0.82 |
| VP1 721-740 | TSTNPSVFW | HLA-B*58:01 | 9 | 0.01 |
|  | TSTNPSVFW | HLA-B*57:01 | 9 | 0.03 |
|  | QTSTNPSVF | HLA-B*46:01 | 9 | 0.16 |
|  | TSTNPSVFW | HLA-B*53:01 | 9 | 0.21 |
|  | WQTSTNPSV | HLA-B*13:02 | 9 | 0.4 |
|  | QTSTNPSVF | HLA-A*25:01 | 9 | 0.4 |
|  | QTSTNPSVF  QTSTNPSVF  WQTSTNPSV  QTSTNPSVF | HLA-C*03:02  HLA-B*15:02  HLA-A*02:06  HLA-C*12:02 | 9  9  9  9 | 0.42  0.44  0.47  0.51 |
| VP1 771-790 | TLNNMGTLY | HLA-A*29:02 | 9 | 0.01 |
|  | TLNNMGTLY | HLA-B*15:25 | 9 | 0.03 |
|  | YGINTLNNM | HLA-C*12:03 | 9 | 0.07 |
|  | TLNNMGTLY | HLA-A*01:01 | 9 | 0.09 |
|  | TLNNMGTLY | HLA-B*46:01 | 9 | 0.13 |
|  | YGINTLNNM | HLA-C*16:01 | 9 | 0.18 |
|  | TLNNMGTLY | HLA-A*03:01 | 9 | 0.21 |
|  | NNMGTLYAR | HLA-A*68:01 | 9 | 0.34 |
|  | YGINTLNNM | HLA-C*14:02 | 9 | 0.36 |
|  | TLNNMGTLY | HLA-B*35:01 | 9 | 0.42 |
| MHC class II alleles | | | | |
| VP1 681-700 | LELTFVITSTQQPST | HLA-DRB1*04:01 | 15 | 2.2 |
|  | ELTFVITSTQQPSTT | HLA-DRB1*08:02 | 15 | 4.9 |
|  | ELTFVITSTQQPSTT  FDLELTFVITSTQQP | HLA-DRB3*02:02  HLA-DQA1*01:01/  DQB1*05:01 | 15  15 | 6.4  7.95 |
| VP1 721-740 | KVDSYVWQTSTNPSV  SYVWQTSTNPSVFWT | HLA-DRB1*04:01  HLA-DRB3*02:02 | 15  15 | 0.82  1.1 |
|  | SYVWQTSTNPSVFWT | HLA-DRB1*08:02 | 15 | 3.9 |
| VP1 771-790 | NGVYGINTLNNMGTL  GVYGINTLNNMGTLY  VYGINTLNNMGTLYA  YGINTLNNMGTLYAR | HLA-DRB3*02:02  HLA-DRB1*13:02  HLA-DRB1*04:01  HLA-DRB1*01:01 | 15  15  15  15 | 1.7  5.2  5.6  8.2 |
