# Supplementary figures and images for: Identification of Immunogenic Epitopes That Permit the Detection of Antigen-Specific T Cell Responses in Multiple Serotypes of Group B Coxsackievirus Infections
Source: Viruses. 2020 Mar 21;12(3):347. doi: 10.3390/v12030347 (PMC7150766; doi:10.3390/v12030347)

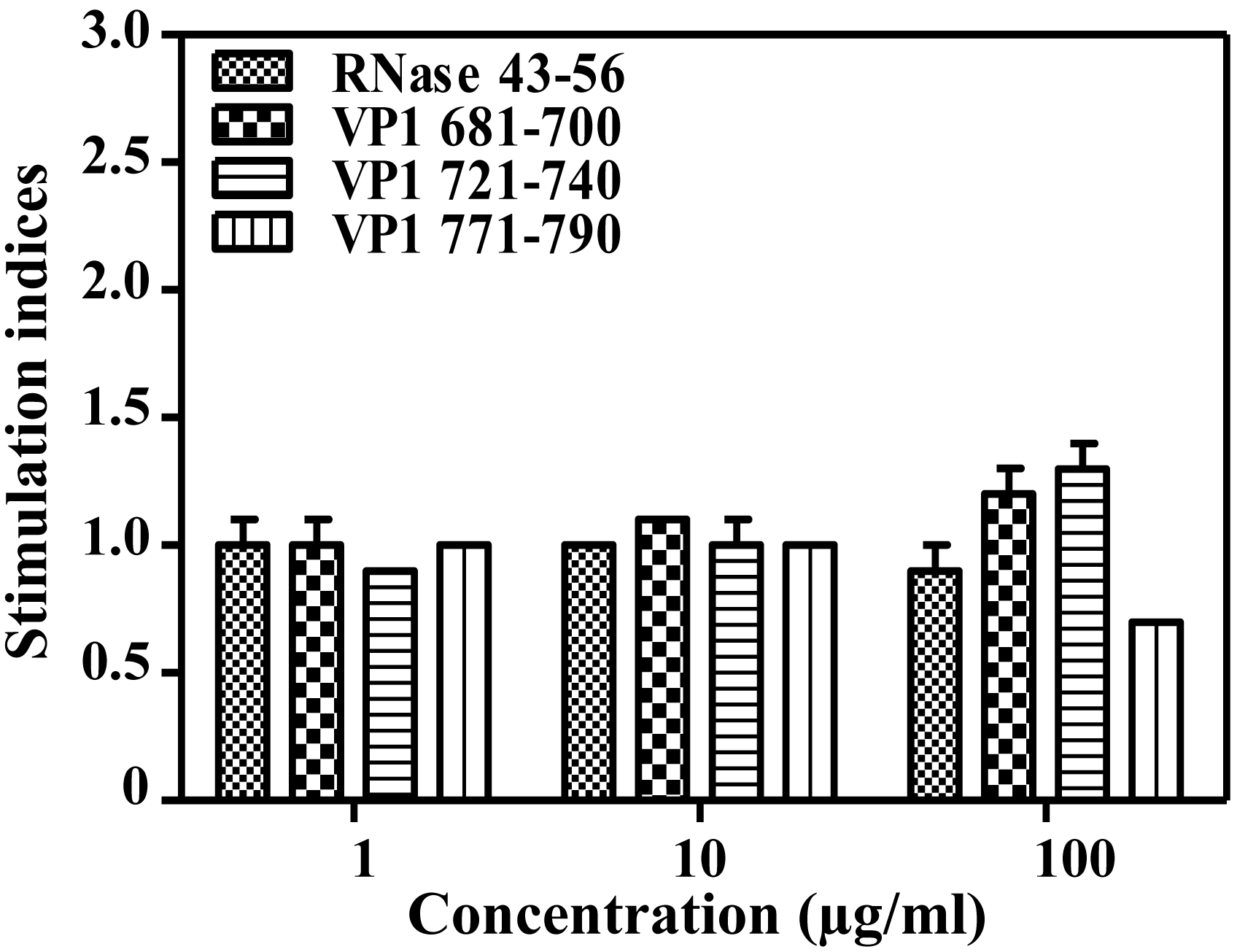

Supplement: Supplementary file 1 [file viruses-12-00347-s001.zip › Supplementary/Supplementary figure 1.tif]

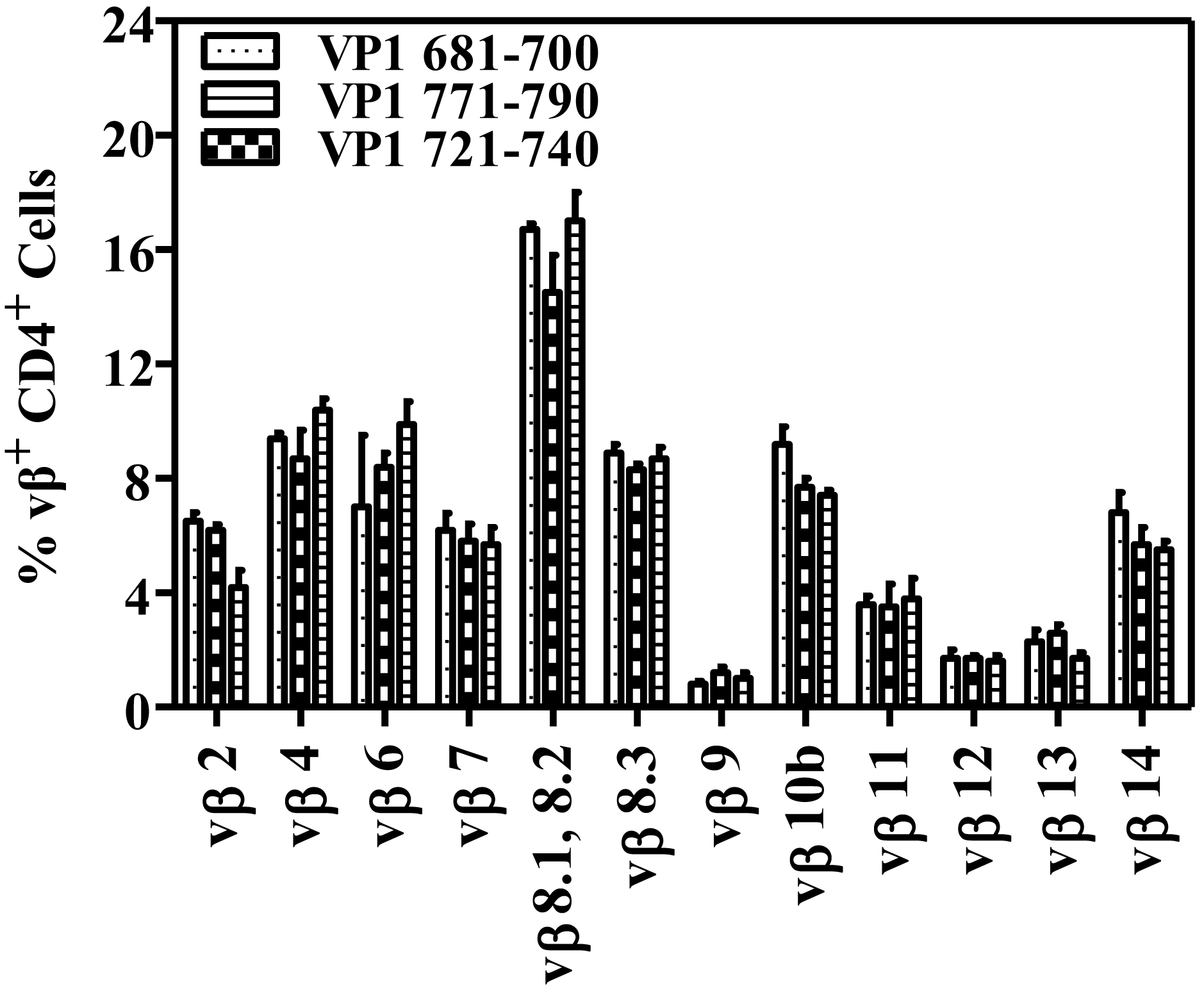

Supplement: Supplementary file 1 [file viruses-12-00347-s001.zip › Supplementary/Supplementary figure 2.tif]

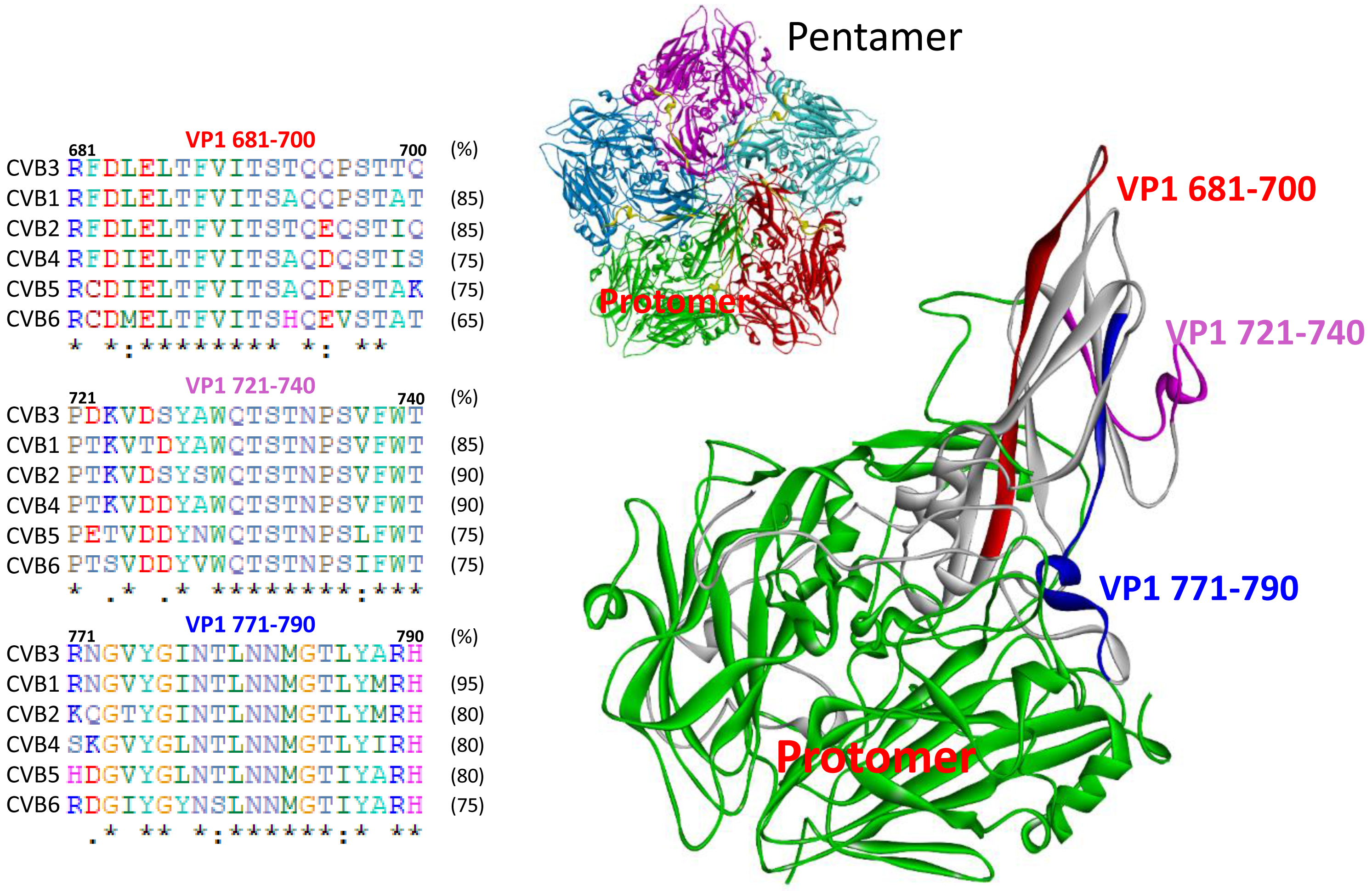

Supplement: Supplementary file 1 [file viruses-12-00347-s001.zip › Supplementary/Supplementary figure 3.tif]
